# Farnesylated and methylated KRAS4b: high yield production of protein suitable for biophysical studies of prenylated protein-lipid interactions

**DOI:** 10.1038/srep15916

**Published:** 2015-11-02

**Authors:** William K. Gillette, Dominic Esposito, Maria Abreu Blanco, Patrick Alexander, Lakshman Bindu, Cammi Bittner, Oleg Chertov, Peter H. Frank, Carissa Grose, Jane E. Jones, Zhaojing Meng, Shelley Perkins, Que Van, Rodolfo Ghirlando, Matthew Fivash, Dwight V. Nissley, Frank McCormick, Matthew Holderfield, Andrew G. Stephen

**Affiliations:** 1 Cancer Research Technology Program, Frederick National Laboratory for Cancer Research, Leidos Biomedical Research, Inc. PO Box B, Frederick, MD 21702; 2 Laboratory of Molecular Biology, National Institute of Diabetes and Digestive and Kidney Diseases, 5 Memorial Drive, Bethesda MD 20892; 3 Data Management Systems, NCI at Frederick, PO Box B, Frederick, MD 21702.

## Abstract

Prenylated proteins play key roles in several human diseases including cancer, atherosclerosis and Alzheimer’s disease. KRAS4b, which is frequently mutated in pancreatic, colon and lung cancers, is processed by farnesylation, proteolytic cleavage and carboxymethylation at the C-terminus. Plasma membrane localization of KRAS4b requires this processing as does KRAS4b-dependent RAF kinase activation. Previous attempts to produce modified KRAS have relied on protein engineering approaches or *in vitro* farnesylation of bacterially expressed KRAS protein. The proteins produced by these methods do not accurately replicate the mature KRAS protein found in mammalian cells and the protein yield is typically low. We describe a protocol that yields 5–10 mg/L highly purified, farnesylated, and methylated KRAS4b from insect cells. Farnesylated and methylated KRAS4b is fully active in hydrolyzing GTP, binds RAF-RBD on lipid Nanodiscs and interacts with the known farnesyl-binding protein PDEδ.

KRAS is a small guanine-nucleotide binding protein which plays a major role in signal transduction leading to growth regulation in cells. Point mutations in *KRAS* are drivers in more than 30% of human cancers, including nearly all pancreatic carcinomas and a large percentage of lung and colorectal tumors^1^. KRAS4b is the primary isoform in human cells, and is post-translationally modified via the CaaX prenylation pathway to permit its interaction with the plasma membrane where much of the signaling process occurs (Figs 1a). These modifications involve an initial cytoplasmic addition of a 15-carbon farnesyl group to Cys185 which is catalyzed by a dual subunit protein called farnesyltransferase (FNT). After prenylation, the protein moves to the surface of the endoplasmic reticulum where it is acted upon by Ras converting enzyme (RCE1) which removes the 3 C-terminal residues of the protein, and then processing is completed by methylation of the new C-terminal farnesylcysteine residue by another ER membrane protein, isoprenylcysteine methyl transferase (ICMT). In the absence of these modifications, KRAS is unable to interact with the membrane, and cannot carry out the necessary protein-protein interactions for proper signaling.

While KRAS has been studied for over 30 years, much of the biochemistry and structural biology of the protein has been carried out using truncated versions of the protein lacking the C-terminus, or full-length unprocessed versions of the protein produced in bacteria. To date the yields and quality of processed KRAS4b protein have been insufficient for structural studies or drug screening experiments, or have failed to recapitulate the full length mature native protein^3^,^4^. In order to fully understand the structure and function of KRAS4b in its native environment, a high yield and high quality method for production of processed protein is essential. To this end, we have developed an engineered baculovirus-based insect cell expression system and purification method that can produce highly purified, fully processed KRAS4b (KRAS4b-FME) at protein levels of 5–10 mg/liter of insect cell culture. The protein was shown to be properly processed using mass spectrometry, and analytical methods were used to demonstrate purity and monodispersity. The protein was shown to bind guanine nucleotides, catalyze nucleotide hydrolysis, and bind in a GTP-dependent fashion to the Ras binding domain (RBD) of CRAF as expected^5^. Further, we demonstrate the ability of processed KRAS4b to interact with lipid Nanodiscs^6^ and the farnesyl binding protein, the delta subunit of retinal rod cGMP phosphodiesterase (PDEδ)^7^, suggesting that the protein is able to mimic essential *in vivo* activities of KRAS.

## Results

### Improving production yield of KRAS4b

The maltose-binding protein (MBP) has been used to enhance the solubility of many proteins expressed in *E. coli*^8^. We have found that MBP also functions as an expression/solubility tag in eukaryotic expression systems (manuscript in preparation) and have inserted it between the His6 tag and the KRAS4b coding sequence in a standard baculovirus expression vector construct. A tobacco etch virus (TEV) protease site (tev) was engineered between the tag and KRAS4b to allow subsequent tag removal during purification. His6-MBP-tev-KRAS4b baculovirus constructs were used to produce baculovirus and to infect two insect cell strains commonly used for protein expression, Sf9 (*Spodoptera frugiperda*) and Hi5 (*Trichoplusia ni*). Protein yields in Sf9 cells were low, but Hi5 cells expressed large quantities of soluble KRAS fusion protein. This protein was readily purified, cleaved with TEV protease, and further purified to remove the solubility tag and protease. Mass spectrometric analysis of this protein showed a single species of 21,551 daltons, corresponding to full length KRAS4b that had not been processed.

### Coexpression of processing enzymes

Although insect cells contain homologs of the two subunits of farnesyltransferase (FNTA and FNTB), it is possible that either of these proteins may not be in high enough abundance to farnesylate all of the KRAS protein, or that the insect versions of these proteins had low activity on the human KRAS4b protein. Most insect Ras proteins use the alternative geranylgeranylation pathway to introduce a 20-carbon prenyl group^9^, and it is possible that insect cells are naturally limited in their capability to farnesylate proteins. In order to overcome this, a baculovirus that coexpressed the two human FNT subunits from two different viral late promoters was generated. Coinfection of this virus with the His6-MBP-tev-KRAS4b virus was carried out and SDS-PAGE analysis demonstrated expression of two proteins corresponding to FNTA and FNTB (data not shown). When KRAS protein was purified from these samples and analyzed, two peaks were observed in intact mass experiments: 21,412 and 21,426 daltons (Supplementary Figure 1). A mass of 21,412 Da is consistent with KRAS4b which has been farnesylated (addition of 204 Da) and proteolyzed by RCE1 (loss of 343 Da), while the larger mass is consistent with addition of a methyl group to the carboxyterminus of the processed protein. No species was observed at the size previously observed for unprocessed KRAS4b (21,551 Da), suggesting that the overexpression of FNT was able to completely farnesylate all of the recombinant protein.

### Baculovirus genome engineering

In order to improve protein yields and reduce the need for virus coexpression, the baculovirus genome was engineered to produce human FNT. The baculovirus genome was present on a single copy *E. coli* plasmid and thus could be readily manipulated by recombineering processes well established in *E. coli* (Supplementary Figure 2). An engineered baculovirus was constructed where a non-essential and potentially deleterious region for protein expression was replaced by the FNTA/FNTB cassette that was previously developed for coinfection experiments. This baculovirus was then modified to generate a His6-MBP-tev-KRAS4b baculovirus and protein was produced and purified as before. As was observed previously, all of the KRAS4b protein was processed, although some unmethylated material remained. However, yields of KRAS4b were improved, presumably because the insect cells were infected with a single virus instead of requiring two viruses. Overall, when using this engineered construct, fully processed (farnesylated, proteolyzed, and methylated) KRAS4b can be isolated with yields of 5–10 mg/liter. This comprises ~70% of the total purified recombinant protein, with KRAS4b-FARN (farnesylated but not methylated) making up the remaining 30%.

### Purification optimization and KRAS4b-FME characterization

**Figure 1:**
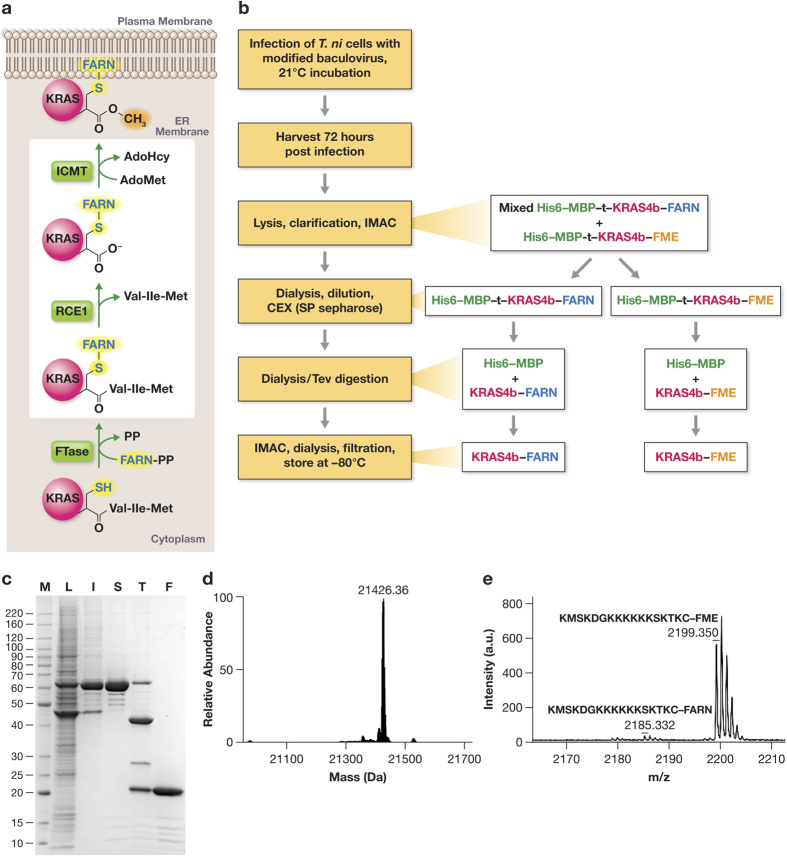
Production of farnesylated and methylated KRAS4b. (**a**) Pathway of KRAS4b processing in eukaryotic cells. Farnesyl pyrophosphate (FARN-PP) is transferred to Cys185 of KRAS4b by farnesyl transferase (FNT; composed of subunits A and B). After transport to the cytoplasmic face of the ER membrane, the 3 amino acids at the C-terminus of KRAS4b are removed by Ras converting enzyme 1 (RCE1) and the terminal carboxylate is methylated by isoprenylcysteine carboxyl methytransferase (ICMT) in a reaction that uses S-adenosylmethionine (AdoMet) and produces S-adenosylhomocysteine (AdoHcy). After methylation, the fully processed KRAS is trafficked to the cytoplasmic face of the plasma membrane. (**b**) Purification scheme of processed KRAS4b using *Trichoplusia ni* (T. ni) insect cells as the expression host. (**c**) SDS-PAGE analysis of purification. M – molecular weight standards; L – soluble lysate; I – pool from initial IMAC; S – SP sepharose pool; T – Tev protease digestion; F – Final protein from second IMAC. (**d**) ESI-MS analysis of final protein (**e)** MALDI-TOF MS/MS analysis of peptides derived from GluC-digested processed KRAS4b confirming the C-terminal peptide is farnesylated and methylated.

The His6-MBP-tev-KRAS4b fusion protein was readily purified by immobilized metal affinity chromatography (IMAC) from the insect cell lysate, however electrospray ionization-mass spectrometry (ESI-MS) analysis of samples from intermediate steps of the process as well as the final purified protein indicated the presence of proteolysis products (largely in the hypervariable region (HVR) between residues 180–184). Additionally, there was evidence of significant loss of processed KRAS during the final size exclusion chromatography (SEC) step (data not shown). Introduction of a cation-exchange step to the process immediately after the initial IMAC essentially eliminated the proteolysis problem and also enhanced the separation of KRAS4b-FARN from KRAS4b-FME (Fig. 1b and Supplementary Figure 3). The loss of processed KRAS to the SEC resin proved to be a problem for all of the resins tested (SEC and desalting). The loss could be reduced to some degree by the use of detergents (e.g. Triton X-100), but as this posed problems for some downstream applications we eliminated the SEC step and achieve buffer exchange by dialysis. SDS-PAGE analysis of the purified KRAS4b-FME indicated the protein was highly pure (Fig. 1c). Intact mass spectrometry analysis of this material indicated that the protein had a mass of 21426 Da consistent with modification by farnesylation and methylation (Fig. 1d). To demonstrate that this additional mass was indeed due to processing at Cys185 we performed Glu-C peptide mapping and MALDI-TOF MS/MS. Proteolysis after Glu168 should generate the C-terminal peptide (KMSKDGKKKKKKSKKTKC with Cys modified by farnesyl and methyl groups; m/z 2199.381), a peptide was identified by MALDI-TOF with a m/z of 2199.350 (Fig. 1e). This peptide was analyzed by MS/MS and both N-terminal *b* - and C-terminal *y*–ions were identified confirming the peptide identity (Supplementary Figure 4). In addition a minor peak was observed with an m/z of 2185.322 consistent with a farnesylated but non methylated peptide in the MALDI analysis (Fig. 1e). Assuming equivalent ionization efficiencies the ratio of the farnesylated and methylated peptide to the farnesylated peptide was 21:1 confirming the predominant species was the fully mature farnesylated and methylated form of KRAS4b.

The secondary structure of KRAS4b-FME was similar to that of non-processed KRAS4b as measured by circular dichroism (Supplementary Figure 5). However, the thermal melting temperature of KRAS4b-FME was 45.3 °C, at least 12 °C lower than that of non-processed KRAS4b (T_m_ 57.7 ^ο^C). Similar melting temperatures were obtained using thermofluor measurements and confirmed that the presence of the farnesyl and methyl groups at the C-terminus destabilized the protein (47.2 °C for KRAS4b-FME, 56.3 °C for the non-processed protein; data not shown). Because KRAS4b-FME displayed lower thermal stability the aggregation status of KRAS4b-FME was determined using sedimentation velocity centrifugation and dynamic light scattering (DLS). Sedimentation velocity (Supplementary Figure 6a) data showed a major species at 2.22 ± 0.01 S with an estimated mass of 22.4 ± 2.4 kDa (in excellent agreement with the theoretical mass of 21,425 Da), indicative of a monomer accounting for 86% of the total signal. Analysis by DLS indicated that 97% of the mass was monomeric (Supplementary Figure 6b). These two techniques confirm the majority of the KRAS4b-FME is present as a monomer.

### Membrane binding, GTPase activity and effector interactions of KRAS4b-FME

**Figure 2:**
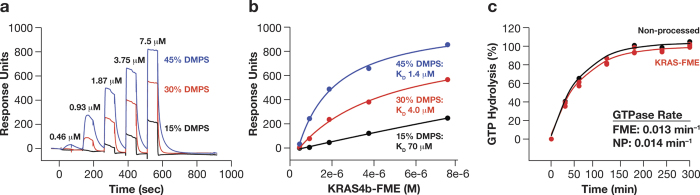
Binding and GTPase activity of KRAS4b-FME on lipid Nanodiscs. The kinetics (**a**) and steady state (**b**) binding of KRAS4b-FME to Nanodiscs composed of variable amounts of DMPS (1,2-dimyristoyl-*sn*-glycero-3-phospho-L-serine) were measured by SPR. (K_D_ values ± SE; 15% DMPS 70 ± 90 μM; 30% DMPS 4.0 ± 0.8 μM; 45% DMPS 1.4 ± 0.3 μM) (**c**) Intrinsic GTPase activity of KRAS-FME or non-processed (NP) KRAS4b on Nanodiscs containing 30% DMPS.

Membrane binding of KRAS4b-FME to lipid Nanodiscs was evaluated by surface plasmon resonance (SPR) spectroscopy. Nanodiscs were prepared using the His6-tagged MSP1D1 protein, 1,2-dimyristoyl-*sn*-glycero-3-phosphocholine (DMPC) and increasing concentrations (15, 30 and 45%) of 1,2-dimyristoyl-*sn*-glycero-3-phospho-L-serine (DMPS). Nanodiscs were captured on sensor chips by amine coupling or by capture using an anti-His6 monoclonal antibody. KRAS4b-FME showed some binding to the carboxymethyl-dextran support on the reference flow cell and this was subtracted from the binding observed on the immobilized Nanodiscs flow cell. No binding was observed to Nanodiscs composed of only DMPC with the KRAS4b-FME concentrations analyzed due to the binding of protein to the reference flow cell and weak binding affinity to Nanodiscs lacking phosphatidylserine. However KRAS4b-FME bound to the Nanodiscs in a phosphatidylserine dependent manner (Fig. 2a,b). The binding kinetics could not be fit to a simple 1:1 binding model. The Nanodiscs were 10 nm in diameter and could accommodate at least two KRAS4b-FME molecules per face; this added complexity may account for the inability of a simple 1:1 binding model to describe the data. The equilibrium binding constants clearly demonstrated that the binding affinity for the Nanodiscs increases increased with increasing phosphatidylserine content (Fig. 2b). Binding of non-processed KRAS4b to the Nanodiscs was not detected in this experimental format (Supplementary Figure 7). In addition the absence of the methyl group also decreased the binding affinity to 30% DMPS Nanodiscs (Supplementary Figure 8; 15.0 ± 1.2 μM (K_D_+/−SE) compared with 4.0 ± 0.8 μM (K_D_+/−SE) for KRAS4b-FME). These data demonstrated that KRAS4b-FME bound with high affinity to lipid membranes such as Nanodiscs. Similar observations have been observed by others with farnesylated and methylated HVR peptides binding to Nanodiscs with different lipid compositions^10^.

The intrinsic GTPase activity of KRAS4b-FME was measured by monitoring the release of soluble ^32^P after hydrolysis of the γ-phosphate bond of ^32^P-GTP. The GDP bound to KRAS4b-FME or non-processed KRAS4b was exchanged with ^32^P-GTP in the presence of an equimolar amount of 30% DMPS containing Nanodiscs (Fig. 2c). The intrinsic GTPase activity of KRAS4b-FME (0.013 +/−0.003 min^−1^) and non-processed KRAS4b (0.014 +/−0.002 min^−1^) in the presence of Nanodiscs were equivalent and are consistent with other published data with non-processed KRAS in the absence of lipid membranes^11^,^12^. These data indicate there was no significant conformational change in the active site of KRAS4b-FME when bound to Nanodiscs that impacts the rate of GTP hydrolysis.

### KRAS4b-FME binds to CRAF-RBD on a Nanodisc

**Figure 3:**
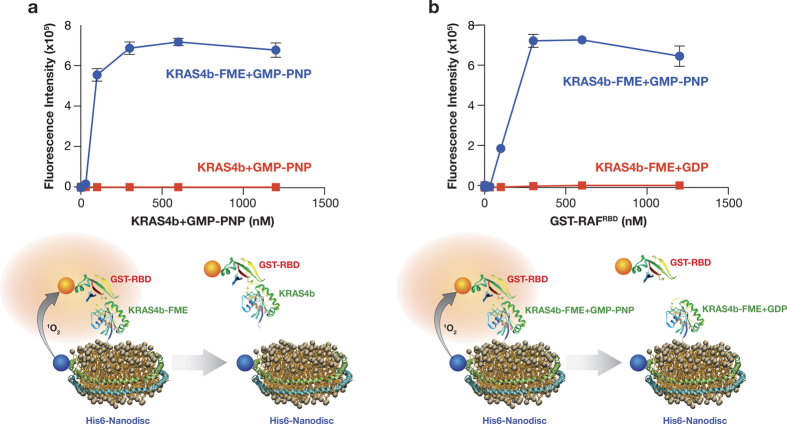
KRAS4b-FME binds to RAF-RBD on a Nanodisc. Binding of KRAS4b-FME to GST-RAF-RBD is detected using the alpha assay. The nickel chelate donor bead recognizes the His6 tag on Nanodiscs (containing 30% DMPS) and transmits a singlet oxygen to the glutathione sepharose acceptor bead that recognizes the GST tag on the RAF-RBD. This alpha signal is observed only with GMP-PNP bound KRAS4b-FME and not non-processed KRAS (**a**). The alpha signal is dependent on the concentration of GST-RAF-RBD and is not observed with GDP bound KRAS-FME (**b**).

Binding of KRAS4b to RAF at the plasma membrane is the first step in signal transduction through the MAPK pathway. We developed an Alpha assay to verify that KRAS4b-FME bound to Nanodiscs was capable of directly interacting with CRAF-RBD. In this assay a nickel chelate donor bead was used to bind the His6 tag on the MSP1D1 protein of the Nanodisc and a glutathione conjugated acceptor bead was used to recognize the GST tag on CRAF-RBD. When the Nanodisc-KRAS4b-FME-RBD complex is assembled, the donor and acceptor beads are brought into proximity, resulting in an increase in the alpha signal (Fig. 3). The alpha signal is only observed in the presence of KRAS4b-FME and not when a non-processed KRAS4b protein is used (Fig. 3a, Supplementary Figure 9), confirming that CRAF-RBD bound to KRAS4b-FME on the surface of the Nanodisc. Binding to CRAF-RBD was concentration dependent and was only observed with KRAS4b-FME bound to the GTP analog GMP-PNP and not GDP (Fig. 3b). Higher concentrations of KRAS4b-FME were required to see binding of CRAF-RBD with Nanodiscs lacking phosphatidylserine compared with those containing 30% phosphatidylserine (data not shown).

### KRAS4b-FME binds to the farnesyl-binding protein PDEδ

**Figure 4:**
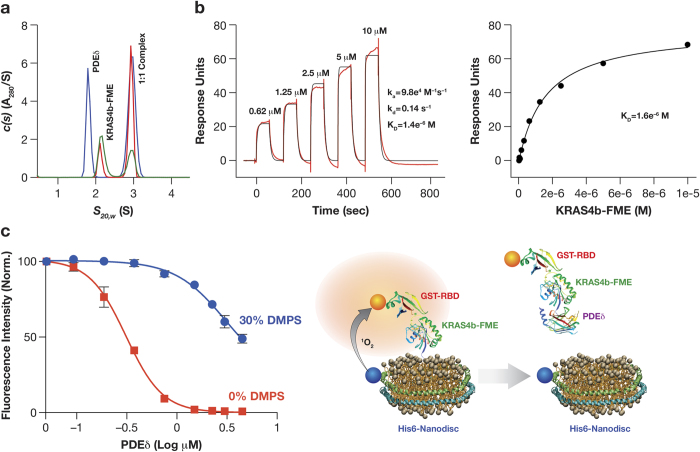
KRAS4b-FME binds to PDEδ. PDEδ and KRAS4b-FME were mixed in ratios of 3:1 (blue), 1:1 (red) and 1:3 (green) and complex formation was measured by analytical ultracentrifugation (**a**). The kinetics (shown in red) of KRAS4b-FME was measured by SPR and could be fit using a simple 1:1 binding model (shown in black) to calculate the microscopic rate constants (**b**, left panel). The steady state binding was fit to determine the equilibrium dissociation constant (K_D_) (**b**, right panel). PDEδ competes for KRAS4b-FME and prevents it binding to Nanodiscs, resulting in detectable fluorescence between the nickel chelate donor and glutathione acceptor beads (**c**).

PDEδ is a farnesyl binding protein that functions as a chaperone during intracellular trafficking of farnesylated RAS and maintains the intracellular spatial organization^13^. Analysis of mixtures of KRAS4b-FME and PDEδ by sedimentation velocity centrifugation indicated a novel species migrating at 2.99 ± 0.02 S (molar mass of 38.0 ± 0.2 kDa) that is consistent with a 1:1 complex of KRAS4b-FME and PDEδ (predicted mass of complex 38.5 kDa, Fig. 4a). No binding of unprocessed KRAS4b to PDEδ was observed (Supplementary Figure 10). Analysis of the binding of KRAS4b-FME to PDEδ amine coupled to an SPR sensor chip could be fit with a 1:1 binding model (Fig. 4b). The equilibrium binding constant was close to 1 μM although the association and dissociation rates were fairly rapid (k_a_: 9.8e^4^ M^−1^s^−1^, k_d_: 0.14 s^−1^). The rapid binding kinetics might be expected for a chaperone protein responsible for moving KRAS4b around intracellular membranes.

Since we showed that binding of KRAS4b to Nanodiscs was dependent on the protein being processed to the FME state (Fig. 3a), we hypothesized that PDEδ might compete with Nanodiscs for KRAS4b-FME binding in the Alpha assay. When KRAS4b-FME was incubated with increasing concentrations of PDEδ prior to the addition of CRAF-RBD and Nanodiscs the signal of the Alpha assay decreased in a concentration dependent manner (Fig. 4c). Interestingly, 10 times higher concentrations of PDEδ were required to prevent KRAS4b-FME from binding to Nanodiscs composed of 30% DMPS (more anionic) than those made exclusively of 100% DMPC (more neutral; Fig. 4c). These results are consistent with the notion that PDEδ competes with lipid bilayers for binding to prenylated proteins, and indicates higher affinity and preferential binding of KRAS-FME to the more anionic Nanodiscs containing phosphatidylserine.

## Discussion

There is emerging evidence that RAS proteins behave differently in the context of a membrane. RAS is only functional in activating CRAF when it is associated with a membrane^14^. HRAS chemically tethered to a lipid bilayer is able to form dimers on membranes but not in solution^15^. NMR studies that suggest KRAS4b tethered to Nanodiscs exists in at least two different orientations and that different effectors alter the orientation of KRAS on the Nanodisc^16^. A deeper understanding of the structure and dynamics of RAS alone or bound with effectors on a membrane will likely provide important molecular details of the mechanisms of signal transduction and also opportunities to develop therapeutic approaches to affect the signaling of oncogenic KRAS. Analysis of processed KRAS4b bound to membranes without chemical tethering likewise should also provide additional insights into the structural dynamics of KRAS4b. In this report we provide a method to generate KRAS4b-FME of sufficient quantity and quality for this type of structural and biophysical analysis.

Previously published approaches for generating lipidated RAS and small GTPase proteins did not recapitulate the native HVR sequence, the prenyl modification found on the terminal Cys *in vivo*, and the methlyation of the Cys residue. Efforts to express and purify processed RAS proteins have had mixed success. Using the insect/baculovirus system, Page *et al.*^17^ reported high expression levels of HRAS leading to the isolation of highly purified palmitoylated (10–20 mg/liter) and non-processed (100–200 mg/liter) protein. Interestingly, expression levels of mutant forms of HRAS^17^ and KRAS^18^ were at least 10-fold reduced from the high levels reported for native HRAS. Lowe and colleagues^4^ describe purification of methylated, farnesylated KRAS at high levels (1.2 mg of protein/4 × 10^8^ cells), although this protein required detergent solubilization and is of limited solubility once purified. However, this group^18^ also reported that the methylation was a limiting factor for both HRAS and KRAS. Campbell and colleagues employed recombinant farnesyl transferase to farnesylate HRAS *in vitro*^19^. A combination of expressed protein ligation and lipopeptide synthesis to generate farnesylated and methylated KRAS4b protein with an additional Cys located between Gly174 and Lys175^20^. A similar approach was taken recently by chemically labelling an HVR peptide with an S-farnesyl-*L*-cysteine methyl ester followed by ligating the peptide to truncated KRAS4b using the bacterial transpeptidase sortase. This process resulted in KRAS4b with additional amino acids LPETG (from the sortase recognition site) inserted between the G-domain and the HVR as well as non-native chemistry in which the farnesyl group and the Cys185 residue were bridged by the bifunctional crosslinking agent SMCC^3^.

In this report we used recombineering to modify a baculovirus to enable production of KRAS4b with the correctly processed HVR at yields of 5–10 mg/L in *T. ni* insect cell culture. This approach benefited from the uniform coexpression of target and modifying enzymes in the infected cells (as opposed to co-infection approaches) and resulted in improved yields (our unpublished observations). Conversely, if the ratio of modifying enzyme to target protein is critical or needs optimization, virus coinfection would seem the preferable approach. The expressed protein was extensively characterized and demonstrated to be farnesylated and methylated, predominantly monomeric and with a secondary structure similar to non-processed KRAS4b (Fig. 1 and Supplementary Figure 5 and 6). Addition of the farnesyl and methyl group to Cys185 decreased the thermal stability of GDP bound KRAS4b-FME by 8–12 ^ο^C (Supplementary Figure 5, and data not shown) compared with full length (2-188) non-processed KRAS4b. During the thermally induced unfolding of KRAS4b-FME the presence of the flexible HVR with an additional hydrophobic moiety may interact with the G domain and destabilize it. It is unlikely that this is biologically relevant as the HVR is constrained by its interaction with the cytoplasmic face of the membrane *in vivo*. However understanding the solution behavior of KRAS4b-FME is important for structural and biophysical analysis.

There is evidence that different RAS isoforms are localized to different domains within the plasma membrane most likely due to alternative prenylation and sequences of the HVRs. HRAS is found predominantly in lipid rafts^21^,^22^ whereas KRAS4b is localized in non-lipid rafts^23^,^24^. The work reported here demonstrates for the first time that recombinant KRAS4b-FME protein binds to Nanodiscs in a phosphatidylserine dependent manner (Fig. 2). Consistent with previous studies, our data demonstrate that KRAS binding to phospholipid requires the farnesyl and methyl group (Supplementary Figure 7), and is additionally enhanced by the electrostatic interaction between the cationic poly-lysine tract and the anionic phospholipids^25^. The absence of the methyl group decreased the affinity to 30% DMPS Nanodiscs, presumably due to the charge repulsion effect of the carboxylic acid group and the slight decrease in lipophilicity of Cys185. The intrinsic GTPase activity of KRAS4b-FME was unaffected when bound to a lipid Nanodisc (Fig. 2) and KRAS4b-FME readily bound CRAF-RBD in a nucleotide-dependent manner on a membrane (Fig. 3). These data validate that the KRAS-FME-Nanodisc complex was catalytically and functionally active. Although both soluble and membrane associated KRAS are capable of binding to effectors, only KRAS-FME in complex with a phospholipid will activate the predominant effector, CRAF^26^. Furthermore, models of membrane tethered KRAS4b suggest it undergoes a conformational change induced by the binding of ARAF-RBD^16^. This observation suggests there may be novel binding pockets that could be exploited with small molecule inhibitors when KRAS4b-FME is bound to RAF-RBD on a membrane. The use of the Alpha assay for CRAF-RBD binding of KRAS4b-FME on Nanodiscs described here provides a high-throughput screening assay platform that may enable the identification of such novel compounds.

Protein-protein interactions that regulate KRAS4b-lipid interactions have been proposed as a novel therapeutic opportunity^27^. There are several reports in the literature that indicate PDEδ plays an active role in maintaining KRAS4b at the plasma membrane^13^,^28^,^29^. Notably, Schmick and colleagues suggest PDEδ plays a role in KRAS trafficking from perinuclear membranes, and PDEδ inhibitors mislocalize KRAS4b to endomembranes^13^. The data presented here indicate that KRAS4b-FME forms a stable complex with PDEδ with a low μM binding constant (Fig. 4). Significantly lower concentrations of PDEδ are needed to prevent KRAS4b-FME from binding to Nanodiscs composed of neutral phospholipids (phosphatidylcholine analog) compared to those with a higher anionic phospholipid component (phosphatidylserine analog; Fig. 4c). These results suggest that KRAS4b-FME has higher affinity for more anionic membranes, and support the role of PDEδ in enhancing the preference for KRAS4b for the more anionic plasma membrane over the much larger surface area of endomembranes in the cell^30^.

In addition to advancing efforts toward therapeutics targeting KRAS-lipid interactions, the techniques described here may be applicable to a number of other prenylated proteins, which may comprise as much as 0.5% of cellular proteins being modified^31^. Prenylated proteins have been implicated in the progression of several human diseases including premature aging^32^, Alzheimer’s^33^, cardiac dysfunction^34^, choroideremia^35^ and cancer^36^. The application of the approach outlined in this work would enable more efficient structural and biochemical analysis of prenylated proteins involved in these diseases and perhaps lead to the identification of novel therapies.

## Materials and Methods

### Preparation of constructs for recombineering

*E.coli* strain DE32 [F- *mcr*A ∆(*mrr-hsd*RMS-*mcr*BC) *φ80*lacZ∆M15 ∆*lac*X74 *rec*A1 *end*A1 *ara*D139 ∆ (*ara*,*leu*)7697 *gal*U *gal*K λ- *rps*L *nup*G/ bmon14272 Δ(*chitinase-cathepsin*); R982-X01 (TetR)] was grown overnight at 37^o^C in 3mL LB with 50 μg/ml Kanamycin. The following day, 2mL of the overnight culture was pelleted and the bmon14272 Δ (*chitinase-cathepsin*) bacmid was purified using a standard alkaline lysis procotol. For bacmid recombineering, the bacmid construct was moved via electroporation into SW106 (generous gift from Don Court, NCI), an *E. coli* strain which has a temperature-inducible lambda prophage integrated into the chromosome. We had previously constructed a Gateway-compatible clone for the bidirectional expression of the two farnesyltransferase subunits (FNTA and FNTB). In this expression clone, FNTA is driven by the baculovirus p10 promoter and FNTB is under the control of the baculovirus polyhedrin promoter. To introduce the genes into the bacmid, we utilized a region of the bacmid which formerly contained the genes for chitinase and cathepsin; this region was previously shown to tolerate insertions^37^,^38^. A linear knock-in cassette was constructed with the isothermal assembly of 5 amplicons including two flanks representing ≈300bp of homology to the regions upstream and downstream from the chitinase-cathepsin deletion, the counter-selectable marker cat-SacB, and both FNT subunits generated from the Gateway expression clone mentioned above (Supplementary Figure 2)^39^. To make the linear cassette, 25 fmoles of each fragment were combined in a 20 ul isothermal assembly reaction with 2× CBA isothermal assembly mastermix (320 μl of 5 × ISO Reaction Buffer [25% polyethylene glycol 8000, 500 mM tris-HCl (pH 8.0), 50 mM MgCl_2_, 50 mM dithiothreitol (DTT), 1.0 μM each dNTPs, 5 μM nicotinamide adenine dinucleotide, 6.4 μl of T5 Exonuclease (1 U/ml) (Epicentre), 20 μl of Phusion polymerase (2 U/ml) [New England Biolabs (NEB)], 80 μl of Taq ligase (40 U/ml) (NEB), and 374 μl of deionized H_2_O^40^. The reaction was incubated at 50 °C for 30 minutes. The cassette was amplified using 1ul of the isothermal assembly reaction, with primers 15165 and 15169 (0.4 μM) in a PCR reaction with 2× Phusion HF mastermix, (100 ul, 25 cycle, 60 °C melting temperature, 6 minute extension) and purified using the Qiagen Qiaquick PCR purification kit. The cat-SacB cassette was amplified from the plasmid template pELO4 (generous gift from Don Court, NCI) in a PCR reaction with 2× Phusion HF mastermix, 0.9 ng template plasmid DNA, 0.4 mM MgCl_2_, and 0.4 μM primers 15173 and 15174 (100 μl, 25 cycle, 62 °C melting temperature, 2 minute extension). The chitinase and cathepsin homology arms were amplified from the bmon14272 Δ(*chi-cth)* bacmid DNA using primers 15165/15347 and 15168/15169 (0.4 μM) in a PCR reaction with 2× Phusion HF mastermix, and 1ul of bacmid template (100 ul, 25 cycle, 60 °C melting temperature, 10 second extension). Primer 15347 has 40 bp of homology to the end of FNTA and the 15168 primer has 40 bp of homology to the end of SacB. The product was purified over a Qiaquick PCR column.

FNTA was amplified from the expression clone R702-M01-624 using oligos 15348 and 15430 0.4 μM in a PCR reaction with 2× Phusion HF mastermix, and 1ng of plasmid template (100 μl, 25 cycle, 60 °C melting temperature, 1 minute extension). Primer 15430 has 40 bp of homology to the beginning of FNTB. FNTB was amplified from the same template using oligos 15429 and 15427 0.4 μM in a PCR reaction with 2× Phusion HF mastermix, and 1ng of plasmid template (100 μl, 25 cycle, 60 °C melting temperature, 1 minute extension). Primer 15427 has 40 bp of homology to the beginning of the cat-SacB cassette. All of the amplicons were purified using Qiagen Qiaquick PCR Purification Kit.

### Baculovirus engineering using recombineering

The SW106 bmon14272Δ(*chi-cth) E.coli* was streaked to LB agar plates with 50 μg/ml Kanamycin. Electrocompetent cells were made from a culture grown to OD600 of ~0.4 that was incubated in a 42 °C water bath with shaking for 15 minutes to induce the lambda Red system^39^. 100 ng of linear FNTA/B-catSacB knock-in cassette was transformed into 50 μl of electrocompetent cells and plated on LB agar with 50 μg/ml kanamycin and 20 μg/ml chloramphenicol. Four colonies were picked and suspended in 50 μl water with vigorous vortexing to mix. 10 μl of sample was removed and heated to 98 °C for 5 minutes in a thermocycler, centrifuged, and 1 μl of crude supernatant was used as template with 0.4 μM primers 15167 and 15353 in a 20 μl reaction with 2× Phusion HF mastermix (30 cycles, melting temperature 60 °C, 30 second extension). Five microliters of this product was electrophoresed on an agarose gel to confirm size. All four colonies were positive for the insertion of the cassette. Five microliters of these diluted cells were then streaked on LB agar with no salt, 6% sucrose, 50 μg/ml kanamycin to test for sucrose sensitivity and all four failed to grow, indicating the proper integration of the cat-SacB cassette. One of the positive colonies was selected, grown and frozen for future use. To remove the cat-SacB cassette, a linear cassette was generated with ≈300 bp of homology to the end of FNTB and ≈300 bp homology to the region downstream of the original cathepsin gene region (Supplementary Figure 2). The end of FNTB was amplified with oligos 15351 and 15349 (0.4 μM) in a PCR reaction with 2× Phusion HF mastermix, and 1ng of R702-M01-624 plasmid template (100 μl, 25 cycle, 60 °C melting temperature, 10 second extension). The downstream cathepsin region was amplified with oligos 15352 and 15169 (0.4 μM) in a PCR reaction with 2× Phusion HF mastermix, and 1 μl of bmon14272 Δ(*chi-cth)* bacmid template (100 μl, 25 cycle, 60 °C melting temperature, 10 second extension). Primer 15352 has 40 bp of homology to the end of FNTB. The two amplicons were attached using overlap PCR with 0.4 μM primers 15351 and 15169 (100 μl, 25 cycle, 60 °C melting temperature, 30 second extension, 0.2 μl of the two amplicons). 100 ng of this marker removal cassette was transformed into 50 μl electrocompetent SW106 bmon14272 Δ(*chi-cth):FNTA-FNTB-catSacB* cells that had been induced for lambda Red recombination. After electroporation, the cells were resuspended in 10mL LB and placed in a 30 °C shaking incubator for 4 hours. A tenth of this culture was pelleted for 30 seconds at 13,000 × g and washed twice with 1× M9 salts. The final pellet was resuspended in 100 μl 1× M9 salts and plated on LB agar with no salt, 6% sucrose, 50 μg/ml Kanamycin. After 36 hours, four colonies were isolated and screened by colony PCR using two sets of primers. The first set (15170 and 15425) to amplify the region of cat-SacB deletion and the second (15167 and 15170) to amplify the entire inserted region. Half of the clones were positive for the correct size products in each case. These two positive clones were streaked on LB agar with 20 g/ml chloramphenicol at 30 °C and neither showed any growth, indicating the loss of the cat-SacB cassette. The remaining diluted cells were used to inoculate 3 mL LB with 50 ug/ml Kanamycin to generate a 15% glycerol stock. From the liquid culture, 2 mL were pelleted for purification of the bmon14272 Δ(*chitinase-cathepsin):FNTA-FNTB* by alkaline lysis. To use the recombineered bacmid in conjunction with the BAC to BAC system, the bmon14272 Δ(*chitinase-cathepsin):FNTA-FNTB* bacmid was moved into a DH10Bac derived strain that contained an optimized helper plasmid R982-X01 (J Mehalko, personal communication). Transformants were plated on LB agar with 50 μg/ml kanamycin and 12.5 μg/ml tetracycline and colonies were screened for the FNTA region by colony PCR using primers 15167 and 15353. A single positive clone was chosen and called DE35 “BAC FNT.” The entire recombined region was amplified using oligos 15167 and 15170, and this amplicon was used as a template to confirm the region by Sanger sequencing.

### Generation of baculovirus expressing KRAS4b

A Gateway Entry clone for human KRAS4b (amino acids 2-188) with an upstream Tev protease cleavage site (ENLYFQG) and three amino acid spacer (GSG) was recombined using Gateway LR Clonase into pDest-636, a pFastBac1 derived Gateway Destination vector containing an amino terminal His6-MBP (maltose-binding protein) fusion tag. The final expression clone was then transformed into *E. coli* DE35 and plated on LB agar with 7 μg/ml gentamicin, 50 μg/ml kanamycin, 10 μg/ml tetracycline, 100 μg/ml Bluo-gal, and 40 μg/ml IPTG. White colonies were grown in liquid culture, and bacmid DNA was purified by alkaline lysis. The junctions of the transposition were screened and a positive clone was used for baculovirus production. Baculovirus was generated and titered as previously described^41^ and was used to infect 1 liter of Sf9 or Hi5 cells for protein expression. Cells were incubated at 21 °C for 72 hours prior to harvest.

### Transfection and expression

General methods for culturing insect cells were described previously^42^ with minor changes as noted here. One day prior to transfection, Sf9 cultures were re-fed to insure that the cells were in logarithmic growth. The day of transfection 100 ml cultures were set in fresh medium amended with 3% FBS (fetal bovine serum) at 1.5 × 10^6^ cells/ml in 490 cm^2^ roller bottles and incubated (27 °C at 100 rpm for 30–60 min) in an INNOVA 4430 shaker) while the transfection mixtures were prepared. Sixty microliters of each bacmid DNA were added to 200 μl of saline in one microcentrifuge tube and 720 μl of transfection reagent (PEI, polyethylenimine^43^) were added to 200 μl of saline in another tube. The diluted bacmid DNAs were added to the diluted transfection reagent and the combinations were mixed gently to avoiding shearing of the bacmid DNA. The mixtures were then incubated for 10 min at room temperature. Each DNA/transfection reagent mixture was added to 100 ml Sf-9 suspension culture and the cultures were immediately returned to the 27 °C shaking incubator. After 4–6 days [see^42^ for critical parameters) the transfected cultures were centrifuged at 2500 rpm, and the supernatants containing the recombinant baculoviruses were saved and stored at 4 °C in the dark. Viral titers of baculovirus stocks were determined using the Sf-9 Easy Titer cells^44^. These supernatants were used as baculovirus stocks in expression experiments. Protein expression was as described previously^42^.

#### Lysis

The cell pellets were thawed and cells were resuspended in a final buffer of 20 mM HEPES, pH 7.3, 300 mM NaCl, 5 mM MgCl_2_, 1 mM TCEP, and 1:100 protease inhibitor (Sigma Aldrich) at a ratio of 100 ml per 1 liter of culture. Cells were lysed mechanically by through a high-pressure instrument (Microfluidizer M-110EH, Microfluidics Corp, Newton, MA). Lysates were clarified by ultracentrifugation (50,000 × g, 30 min) and stored at −80 °C until purification.

#### Purification

Clarified lysate was thawed, adjusted to 35 mM imidazole and loaded at 3 ml/min onto a 10 ml IMAC column (HisTrap, GE Healthcare) per liter of culture. The equilibration buffer (EB) for the column was 20 mM HEPES, pH 7.3, 300 mM NaCl, 5 mM MgCl_2_, 1 mM TCEP, 35 mM imidazole and 1:1000 protease inhibitor cocktail. The column was washed to baseline with EB and proteins eluted with a 20 column-volume (CV) gradient from 35 mM to 500 mM imidazole. Elution fractions were analyzed by SDS-PAGE and Coomassie-staining.

Positive fractions were pooled, dialyzed to 20 mM MES, pH 6.0, 150 mM NaCl, 5 mM MgCl_2_, 1 mM TCEP. Dialyzed sample was centrifuged at 4,000 × g for 15 min to remove precipitate and then diluted 1:1 with dialysis buffer minus NaCl to bring the salt concentration to 75mM immediately prior to loading.

Sample was loaded at 3 ml/min to an SP Sepharose column (identical in size to the initial capture column used in IMAC) equilibrated in 20 mM MES, pH 6.0, 75 mM NaCl, 5 mM MgCl_2_, and 1 mM TCEP. The column was washed to baseline with same buffer and eluted with a 20 CV gradient from 75 mM to 650 mM NaCl. The eluate was collected in fractions (40% of CV). The fractions from appropriate peaks (Supplementary Figure 3) were pooled and Tev protease added at a 1:10 molar ratio. Digest proceeded while dialyzing to 20 mM HEPES, pH 7.3, 300 mM NaCl, 5 mM MgCl_2_, and 1 mM TCEP overnight at 4 °C. The digested sample was processed by IMAC; column equilibration buffer and wash buffer were of the same composition as the dialysis buffer used in the previous step (no imidazole). Column flow through and wash were collected as fractions as a precaution, and the column was developed with a 10 CV gradient to 50 mM imidazole. Appropriate fractions (the target protein typically elutes at ~25% imidazole (approx. 12.5mM imidazole) were pooled, dialyzed, and, if necessary, concentrated in 10K Amicon centrifugation units before creating final aliquots for storage at -80C.

### Molecular weight confirmation of recombinant KRAS4b-FME by LC-MS

Recombinant protein was diluted based on its concentration and 1 μL was injected for accurate intact mass analysis by LCMS using an Agilent 1200 nanoflow LC system coupled online with a LTQ Orbitrap XL mass spectrometer. The RPLC column (75 μm i.d. × 10 cm) was slurry-packed in-house with 5 μm, 300 Å pore size C-4 stationary phase into fused silica capillaries with a flame pulled tip. After sample injection, the column was washed for 30 min with 95% mobile phase A (0.1% formic acid in water) at 0.5 μl/min. Proteins were eluted into mass spectrometer using a linear gradient of 5% mobile phase B (0.1% formic acid in ACN) to 50% B in 30 minutes, then to 80% B over an additional 5 minutes at 0.5 μl/min. ESI-MS spectrum of the protein acquired in profile mode at 60000 resolution was further processed using Xtract function in Thermo Xcalibur Qual Browser at S/N of 10 to generate its molecular weight information.

### Confirmation of farnesylation and methylation at Cys185 by MALDI-TOF MS/MS

2 μL of KRAS4b-FME (1 mg/ml) diluted with 8 μL of buffer containing 20 mM HEPES pH7.3, 100 mM NaCl, 5 mM MgCl_2_, 0.5 mM DTT and added 1 μL of endoproteinase Glu-C dissolved in water at 0.5 mg/mL (Roche, Cat No 11 420 399 01). After incubation for 19 hours at room temperature (24 °C) in the dark 1 μL of the digest was mixed with 10 μL of DHB (2,5-dihydroxybenzoic acid) matrix (saturated in solution containing 10% acetonitrile, 0.1% trifluoroacetic acid), 2 μL of the mixture spotted on target plate and analyzed by matrix-assisted laser desorption ionization time-of-flight mass spectrometry (MALDI-TOF MS) on Ultraflex III mass spectrometer (Bruker Daltonics) in reflector mode with 3000 laser shots acquired per spectrum (Fig. 1d). The m/z 2199.350 which was close to the expected m/z of C-terminal peptide of KRAS4b (KMSKDGKKKKKKSKKTKC) with carboxyl methylated S-farnesyl cysteine residue (expected m/z 2199.381) was analyzed by MALDI-TOF MS/MS. The spectrum information was processed using BioTools resident software (version 3.34) and compared with expected sequence KMSKDGKKKKKKSKKTKC with farnesyl and methyl groups on terminal Cys (C16H27, 218 mass units). Both N-terminal *b* - and C-terminal *y*–ions were identified confirming peptide identity (Supplementary Figure 4).

### CD measurements

WT KRAS was buffer exchanged into 0.1M potassium phosphate, pH 7.3 using a pre-packed PD-10 column as per the manufacturer’s instructions. KRAS4b-FME was buffer exchanged into the same buffer using an Amicon centrifugal filter device 5,000 MWCO at 4 °C with a 30 fold excess of 0.1M potassium phosphate, pH 7.3 than the starting protein volume. Proteins were quantitated by UV absorbance using ε_280_ = 19685 M^−1^cm^−1^. CD spectra were collected on a Chirascan Plus (Applied Photophysics) at 25 °C from 250–190 nm with a 1 nm step and bandwidth, time per point 1 second, 1 mm pathlength, and 0.1 mg/ml protein. Phosphate buffer blank was auto-subtracted from each spectra and three were averaged from which MRE was calculated using the concentration, pathlength, and number of amino acids residues in the polypeptide chain with the program software. Thermal melts of 0.1 mg/ml protein in 0.1 M potassium phosphate pH 7.2 in stoppered 1mm cuvettes were obtained with a continuous temperature ramp of 1 °C min^−1^ controlled by the instrument Peltier heating/cooling unit. Transitions were monitored by the loss of signal at 222 nm, and Tm’s were calculated using the Global3 analysis software provided with the system.

### Dynamic Light Scattering

A 1 mg/ml solution of processed KRAS in 20mM Hepes pH 7.3, 150mM NaCl, 5mM MgCl_2_, 1mM TCEP was centrifuged five minutes 16,000 × g at 4 °C. 35 ul was pipetted into a Corning 3540 384 well assay plate. The plate was centrifuged five minutes 820 × g at 25 °C to remove bubbles. Dynamic light scattering data was collected on a DynaPro Platereader II (Wyatt Technologies) at 25 °C with ten acquisitions per well, and a five second acquisition time.

### Sedimentation velocity

Sedimentation velocity experiments were conducted at 50,000 rpm and 20 °C on a Beckman Coulter ProteomeLab XL-I analytical ultracentrifuge following standard protocols^45^. A sample of 0.6 mg/mL KRAS4b-FME in 20 mM HEPES (pH 7.3), 150 mM NaCl, 5 mM MgCl_2_ and 1 mM TCEP was loaded into 2-channel, 12 mm path-length sector shaped cells and thermally equilibrated at zero speed. Absorbance and interference velocity scans were subsequently acquired at approximately 2.5 minute intervals – absorbance data were collected in a continuous mode as single measurements at 280 nm using a radial spacing of 0.003 cm. Time corrected data were analyzed in SEDFIT 14.4f in terms of a continuous c(*s*) distribution of sedimenting species using an *s* range of 0 to 20 with a linear resolution of 400 and a maximum entropy regularization confidence interval of 0.68. Excellent fits were observed with root mean square deviations of 0.0037 absorbance units and 0.0058 fringes. The partial specific volume of KRAS4b-FME was calculated based on its amino acid composition using SEDNTERP (http://sednterp.unh.edu/); the solution density ρ and viscosity η were also calculated in SEDNTERP based on the composition. Sedimentation coefficients were corrected to standard conditions in water at 20 °C, *s*_*20,w*_. Sedimentation velocity experiments were also carried out to characterize the high affinity PDEδ and KRAS4b-FME complex. Samples of 15 μM PDEδ and 25 μM KRAS4b- FME in 20 mM HEPES (pH 7.3), 200 mM NaCl, 5 mM MgCl_2_ and 1 mM TCEP were characterized as described above, along with their mixtures of approximately 1.8:1, 0.6:1 and 0.2:1 PDEδ:KRAS4b-FME all containing 27 μM KRAS4b. All samples were loaded into 12 mM path-length cells, except for the 1.8:1 mixture which was analyzed in a 3 mM path-length cell. The c(*s*) analyses utilized an s range of 0–5 with a linear resolution of 100 and excellent fits were observed with root mean square deviations ranging from 0.0046–0.0064 absorbance units and 0.0035–0.0052 fringes.

### Nanodisc preparation

The DMPC and DMPS lipids were purchased from Avanti Polar Lipids in chloroform and used without further purification. Lipid concentration was determined by Avanti using total phosphate analysis. Lyophilized MSP1D1 scaffold protein was purchased from Sigma Aldrich and reconstituted in DI water and the concentration was determined by UV absorbance at 280nm. The Nanodiscs were prepared based on literature protocols^6^. DMPC and DMPS stock lipids in chloroform were mixed together in 70:30 DMPC/DMPS ratio and slowly dried using a low flow of argon gas while incubating in a bead heat bath at 55 °C. The dried lipid was reconstituted to 65 mM with 130 mM cholate, 20 mM Tris, 100 mM NaCl, and 0.5 mM EDTA buffer, and mixed with MSP1D1 in a 90:1 lipid to protein ratio. The mixture was mixed on a Nutator for 1 hour, and then 1 g/mL of washed biobeads was added and incubated at RT for an additional 4 hours on a Nutator. Afterwards, the Nanodiscs were removed by careful pipetting and gel filtration chromatography was performed on an AKTA FPLC with a GE Superdex 200 increase column (10 × 300 mm) running at 0.5 mL/min flow rate.

### GTP hydrolysis experiments

The GTPase hydrolysis activity of processed KRAS4b and non-processed KRAS4b were compared in the presence of Nanodiscs. Non-processed wild type KRAS4B (2-188) was expressed and purified from *E. coli* (manuscript in preparation). 5 μg of KRAS4b-FME-GDP or non-processed KRAS4b-GDP was incubated with an equimolar equivalent of Nanodiscs containing 30% DMPS at 30 °C for 15 min in exchange buffer (50 mM Tris-HCl pH7.5, 5mM DTT, 50 mM NaCl and 10 mM EDTA) with 2 μCi of γ-^32^P-labeled GTP in a total volume of 100 μl. 3 μg of each KRAS4b-^32^P-labeled GTP sample was incubated at 37^o^C in 300 μl of hydrolysis buffer supplemented with 1 mM MgCl_2_. Aliquots were removed and added to 400 μl of prechilled stop buffer that consisted of 5% activated (w/v) charcoal, 0.2 M HCl, 1mM NaH_2_PO_4_ and 20% (v/v) ethanol. After centrifugation soluble ^32^P counts are measured by scintillation counting. Observed counts at each time point were converted to a percentage of total counts. Percentage results were modeled with an exponential function; rates and associated variation were extracted using Matlab’s nonlinear regression system.

### SPR measurements

SPR binding experiments were performed on a Biacore T200 instrument (GE, Piscatawy, NJ). Binding measurements of KRAS4b-FME were carried out as follows. Anti-His6 monoclonal antibody (R&D systems) was coupled to the carboxymethylated dextran surface of a CM5 sensor chip (GE, Piscatawy, NJ) using standard amine coupling chemistry. In brief, the surface was activated with 0.1 M N-hydroxysuccinimide and 0.4 M N-ethyl-N’-(3-dimethylaminopropyl) carbodiimide at a flow rate of 20 μl/min. Anti-His6 Mab was diluted to 20 μg/ml in 10 mM sodium acetate (pH 5) and injected on all 4 flow cells until a density of approximately 5000 RU was attached. Activated amine groups were quenched with an injection of 1 M ethanolamine (pH 8.0). 600-800 RU of Nanodiscs composed or 15, 30 or 45% DMPS were captured on flow cells, 2, 3 and 4; flow cell 1 was used for referencing purposes. After Nanodisc capture a series of buffer injections were performed in the running buffer [20 mM HEPES (pH 7.5), 150 mM NaCl] to establish a stable baseline. KRAS4b-FME or non-processed KRAS4b was diluted in running buffer, to concentrations indicated in the figure legends and injected over the captured Nanodiscs for 1 min at 30 μl/min at 25 °C. At the end of the injection, bound KRAS4b-FME was removed by a 60 sec injection of 100 mM phosphoric acid. Experiments were also performed where Nanodiscs were amine coupled directly to the CM5 chip using standard amine coupling chemistry and the binding of KRAS4b-FMEwas measured. The binding of KRAS4b-FME was comparable when Nanodiscs were captured or directly coupled to the chip surface. Binding measurements of KRAS4b-FME to PDEδ were carried out as follows. PDEδ was amine coupled to flow cells 2 and 4 to a density or 200 or 400 RU as outlined above. KRAS4b-FME or non-processed KRAS4b was diluted in triplicate in running buffer [20 mM HEPES (pH 7.5), 150 mM NaCl, 100 mM TCEP] to concentrations indicated in the figure legends and injected over the PDEδ for 1 min at 30 μl /min at 25 °C. The PDEδ surface was regenerated using 100 mM phosphoric acid. Equilibrium or kinetic data was fit using the Biacore Evaluation software to determine the binding constants.

### KRAS4b-RAF-RBD biochemical binding assay

The *in vitro* protein-protein interaction between wild-type KRAS4b (FME expressed in the baculoviral system described above or Avi-tagged-KRAS4b expressed in *E. coli*, which lack the farnesyl transferase and ICMT enzymes necessary for complete processing of the C-terminus) and GST-RAF-RBD was determined by measuring binding using PerkinElmer’s AlphaLISA Technology. (His6)-Nanodisc (0 or 30% DMPS) and KRAS4b (FME or bacterially expressed Avi-tagged KRAS) were mixed at the desired molar ratio in 20 mM Tris (pH 7.5), 100 mM NaCl, 0.5 mM EDTA and 0.1 mM DTT; the mix was incubated for 30 min at room temperature in a nutator. KRAS4b nucleotide loading was performed by diluting KRAS-FME or Avi-KRAS to 10 μM in Nucleotide Exchange Buffer (50 mM Tris pH 7.5, 5 mM NaCl, and 0.1 mM DTT), with 100 μM GMP-PNP (Jena Bioscience) or 1 M GDP (Sigma-Aldrich) accordingly. Loading reactions were incubated for 15 min at 37 °C, then a 20x molar excess of MgCl_2_ to EDTA was added to the reaction. KRAS-CRAF-RBD binding reactions were carried out by mixing (His6)-Nanodisc-KRAS4b (FME or biotinylated KRAS) and GST-RAF-RBD (1200-0 nM final concentration) in 50 mM Tris (pH 7.5), 5 mM NaCl, and 0.1 mM DTT; the reactions were incubated for 1 hour at room temperature. A 30 μl mix of Nickel Chelate Alpha Donor beads (PerkinElmer) and Glutathione Acceptor beads (PerkinElmer) in 50 mM Tris (pH 7.5) was added to each reaction and the mix was incubated for 1 hour at room temperature and protected from light. The reactions were read on a PerkinElmer EnVision® Multilabel Reader. All assays were performed in 384-well LIA-plates (Grenier Bio-0ne) by triplicate. For competition/displacement assays with PDEδ, the assay was performed as described above with one modification: prior to (His6)-Nanodisc binding, PDEδ and KRAS4b-FME were mixed at the desired ratio, and incubated for 10 minutes at room temperature in the nutator.

## Additional Information

**How to cite this article**: Gillette, W. K. *et al.* Farnesylated and methylated KRAS4b: high yield production of protein suitable for biophysical studies of prenylated protein-lipid interactions. *Sci. Rep.* **5**, 15916; doi: 10.1038/srep15916 (2015).

## Supplementary Material

Supplementary Figures
