# Supplementary material for: Farnesylated and methylated KRAS4b: high yield production of protein suitable for biophysical studies of prenylated protein-lipid interactions
Source: Sci Rep. 2015 Nov 2;5:15916. doi: 10.1038/srep15916 (PMC4629113; doi:10.1038/srep15916)
Supplement: Supplementary Figures [file srep15916-s1.pdf]

Farnesylated and methylated KRAS4b: high yield production of protein suitable for biophysical studies of prenylated protein-lipid interactions

William K. Gillette[1], Dominic Esposito[1], Maria Abreu Blanco[1], Patrick Alexander[1], Lakshman Bindu[1], Cammi Bittner[1], Oleg Chertov[1], Peter H. Frank[1], Carissa Grose[1], Jane E. Jones[1], Zhaojing Meng[1], Shelley Perkins[1], Que Van[1], Rodolfo Ghirlando[2], Matthew Fivash[3], Dwight V. Nissley[1], Frank McCormick[1], Matthew Holderfield[1] and Andrew G. Stephen[1]*

[1]Cancer Research Technology Program, Frederick National Laboratory for Cancer Research, Leidos Biomedical Research, Inc. PO Box B, Frederick, MD 21702. [2]Laboratory of Molecular Biology, National Institute of Diabetes and Digestive and Kidney Diseases, 5 Memorial Drive, Bethesda MD 20892. [3]Data Management Systems, NCI at Frederick, PO Box B, Frederick, MD 21702.

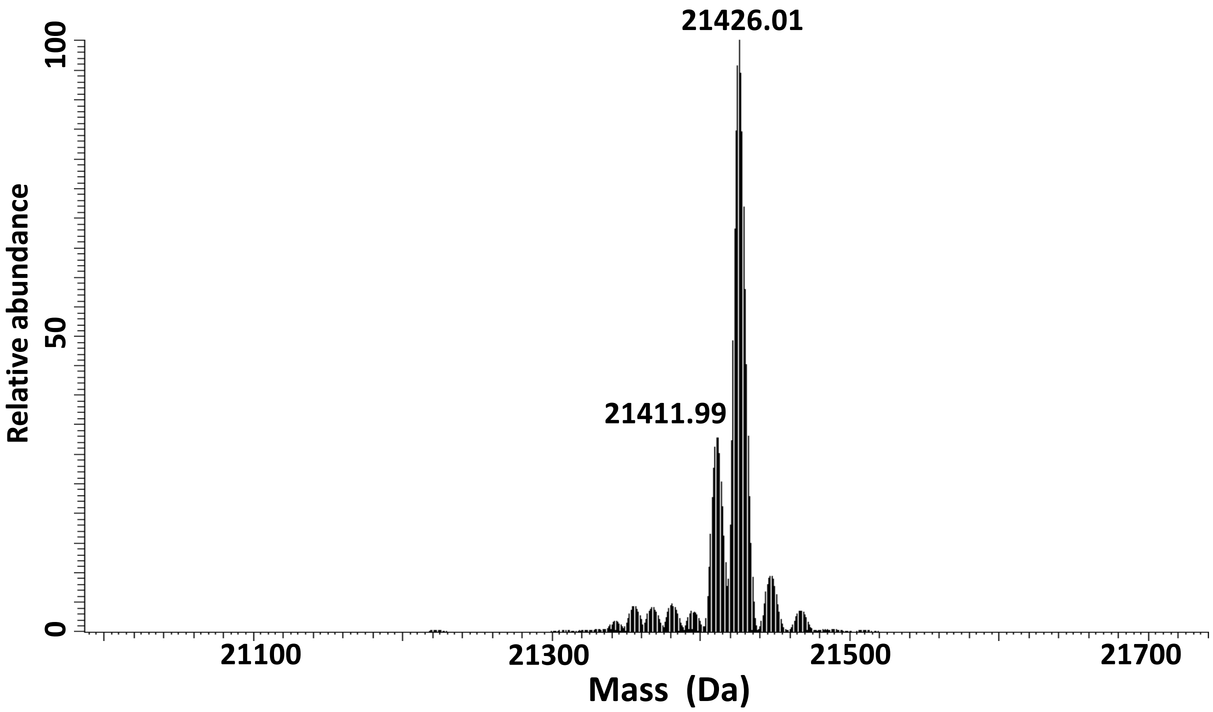

Figure S1: ESI-MS detection of a mixed population of processed KRAS4b purified from insect cells.

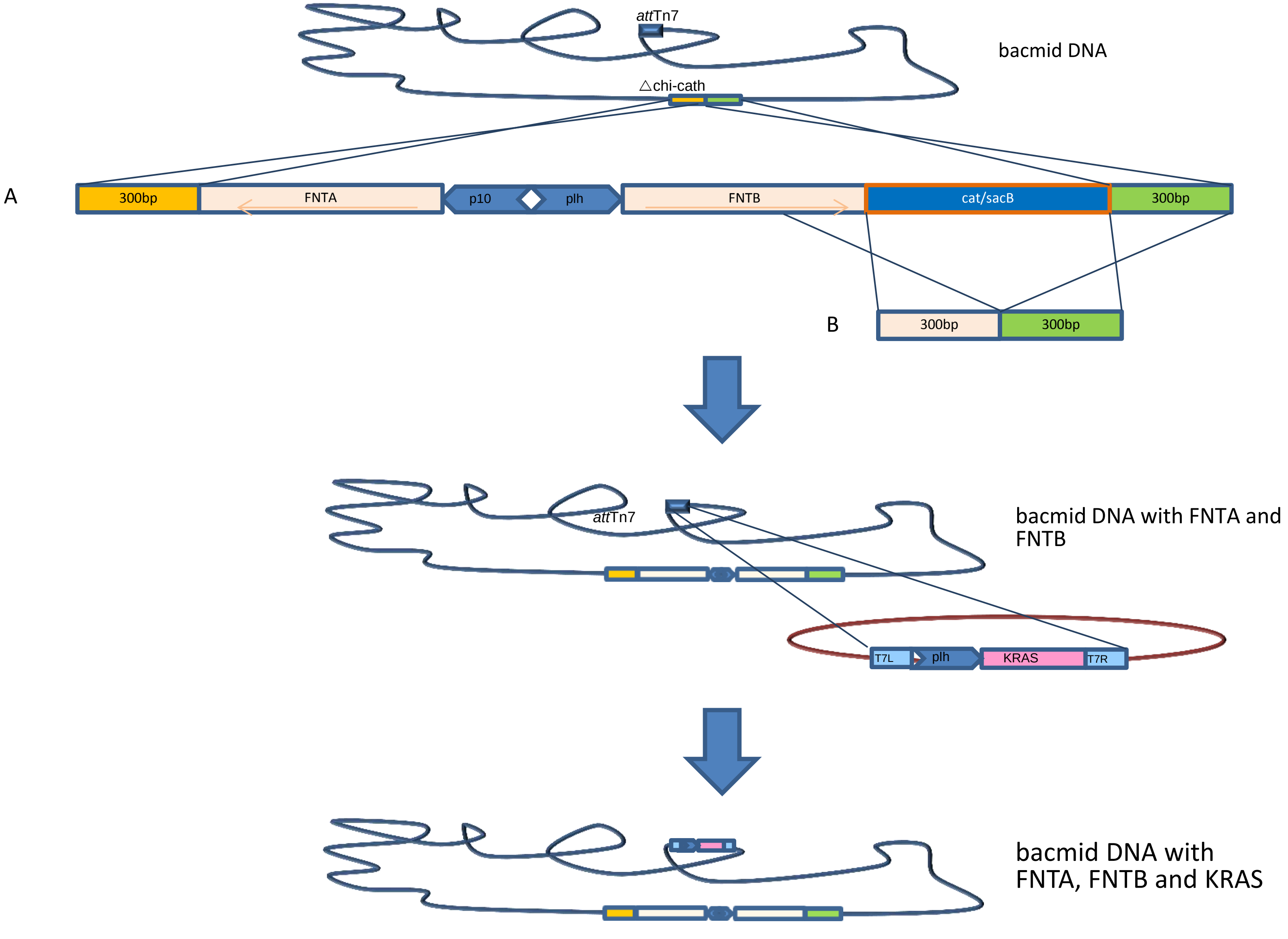

Figure S2: Strategy for engineering the baculovirus genome for prenylated protein production.

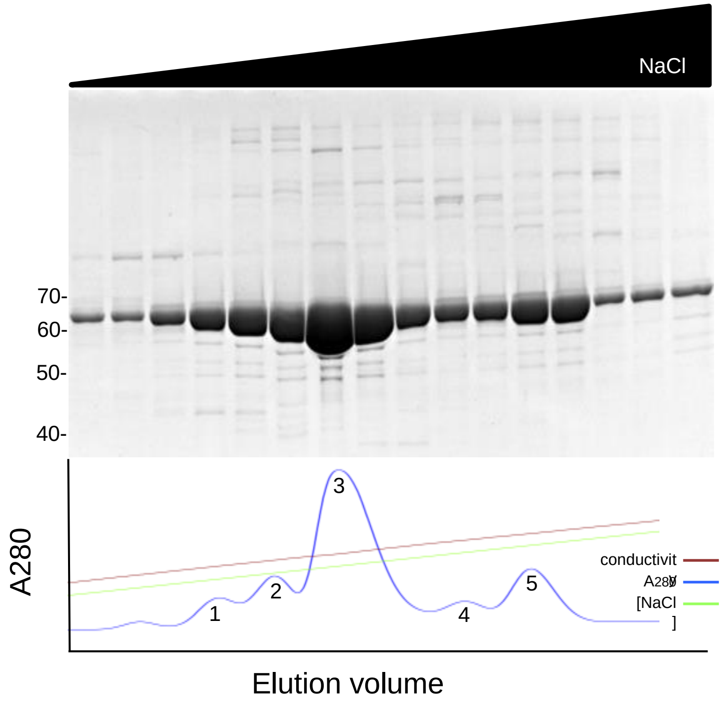

Peak	1	2	3	4	5
ESI-MS (Da)	20976	21412	21426	21412<br>20975	21426
KRAS	T183*	FARN	FME	FARN<br>T183*	FME

Figure S3: Separation of KRAS-FME from KRAS-FARN using ion exchange chromatography.

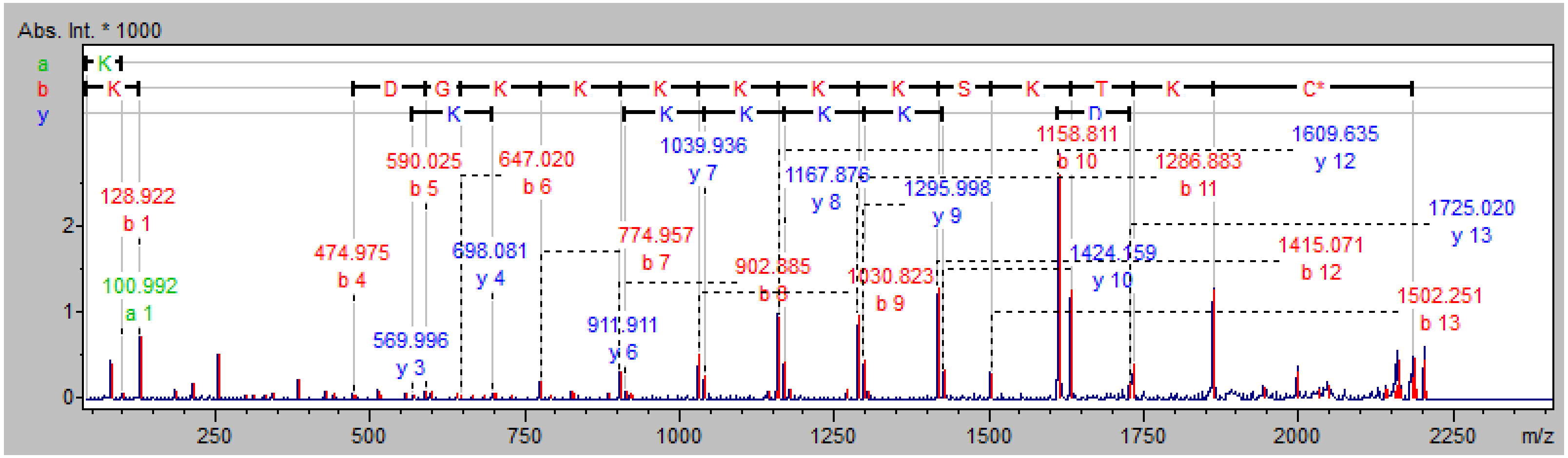

Figure S4: MALDI-TOF MS/MS analysis of m/z 2199.350

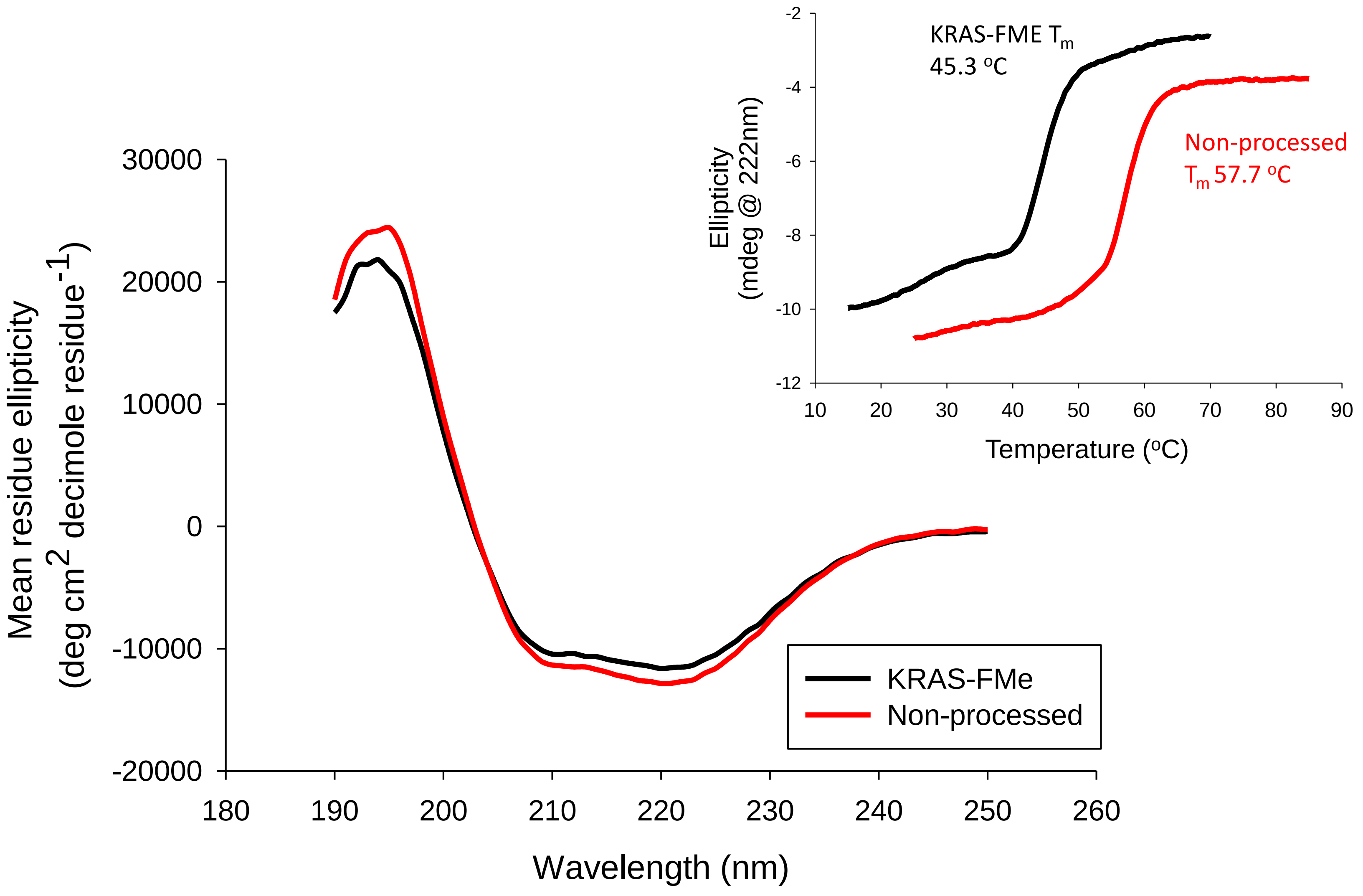

Figure S5: Circular dichroism spectra of non-processed and KRAS4b-FME.

a

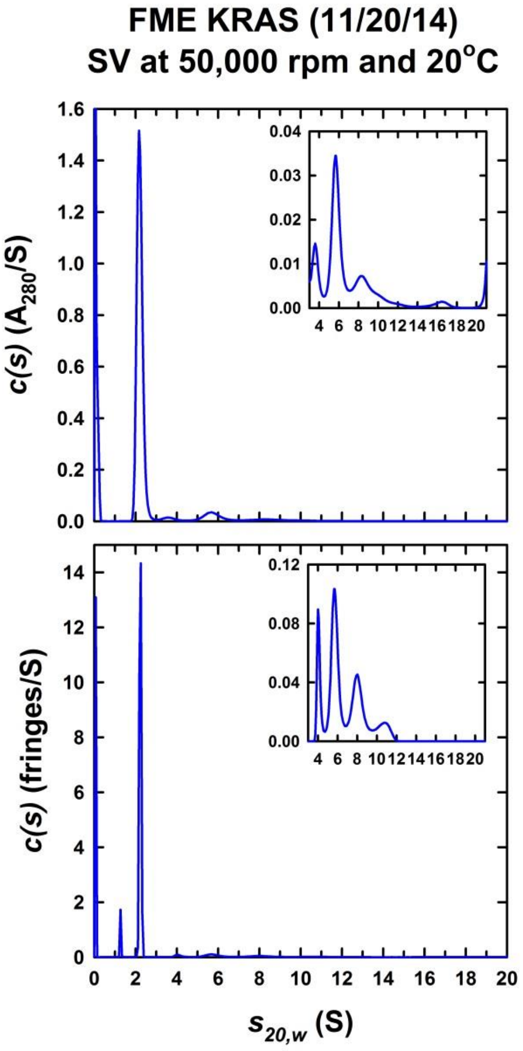

b

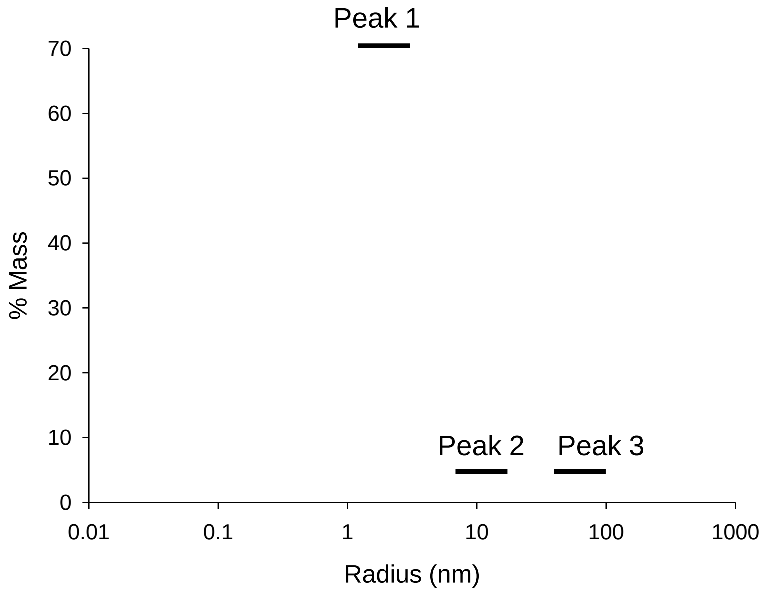

	Radius (nm)	Polydispersity %	Molecular weight (kDa)	Intensity %	Mass %
Peak 1	1.9	15.1	16	1.6	96.7
Peak 2	12.8	21.8	1311	7.8	1.7
Peak 3	89.5	32.9	124311	90.6	1.6

Figure S6: AUC and DLS data indicates KRAS4b-FME is predominantly monomeric.

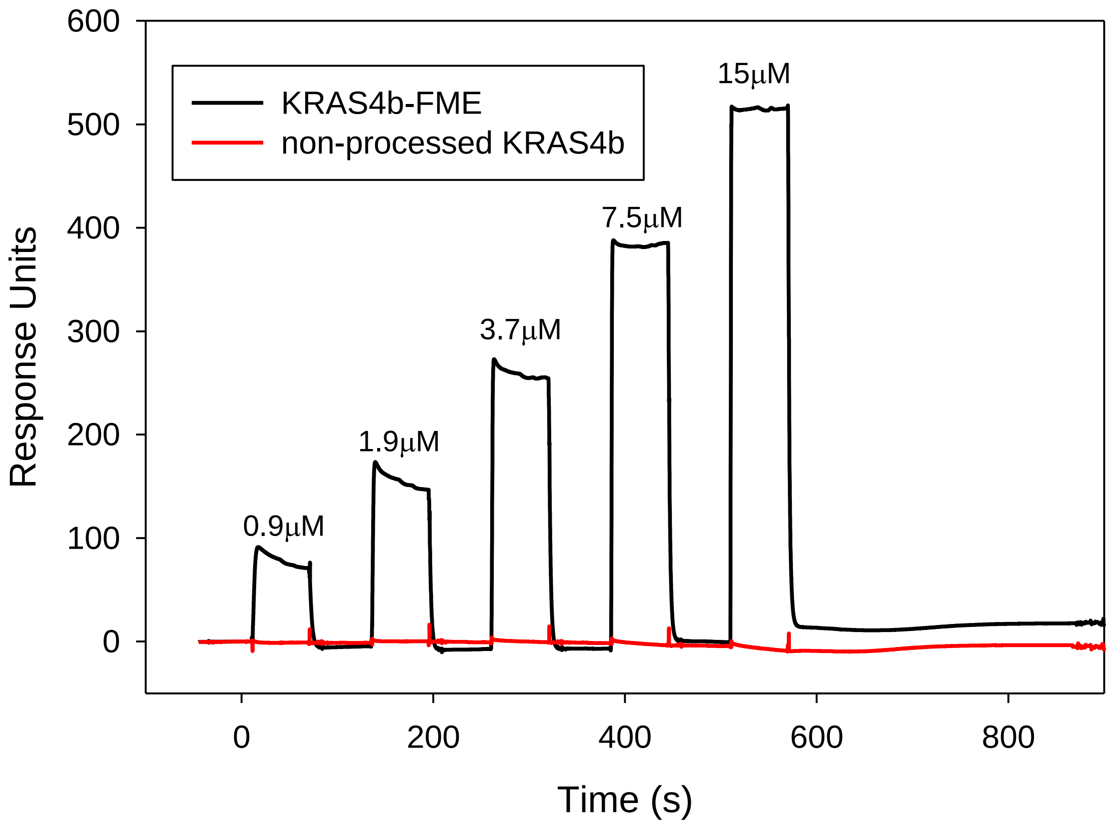

Figure S7: Non-processed KRAS4b does not bind to Nanodiscs.

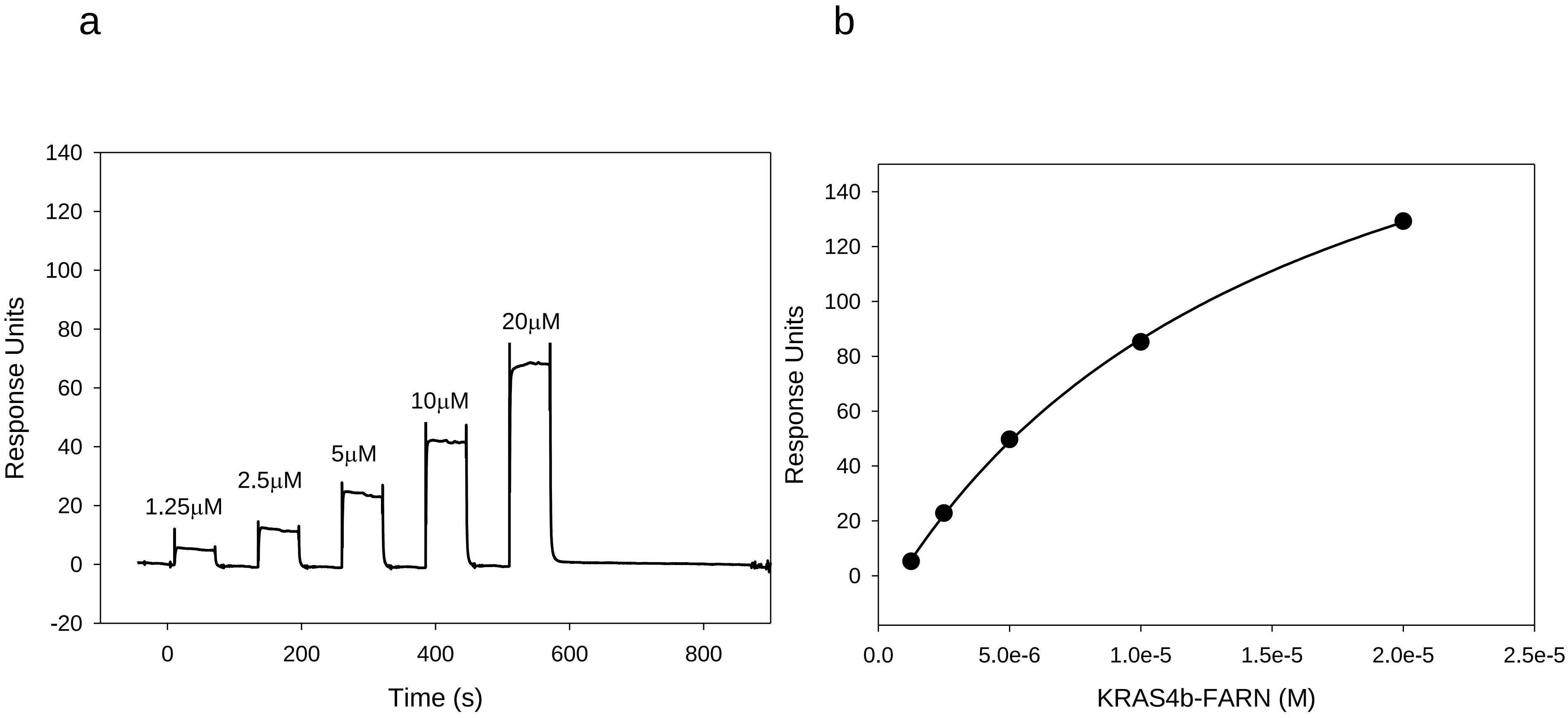

Figure S8: Farnesylated KRAS4b binding to 30% DMPS Nanodiscs

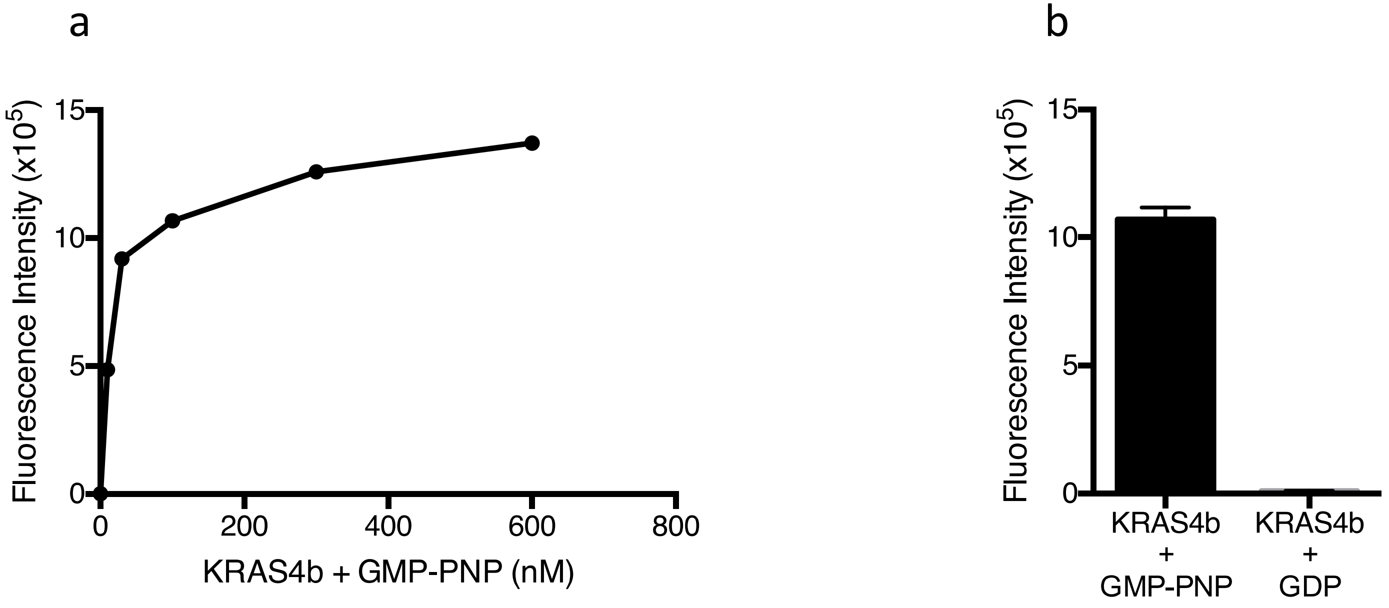

Figure S9: KRAS4b binds to CRAF-RBD.

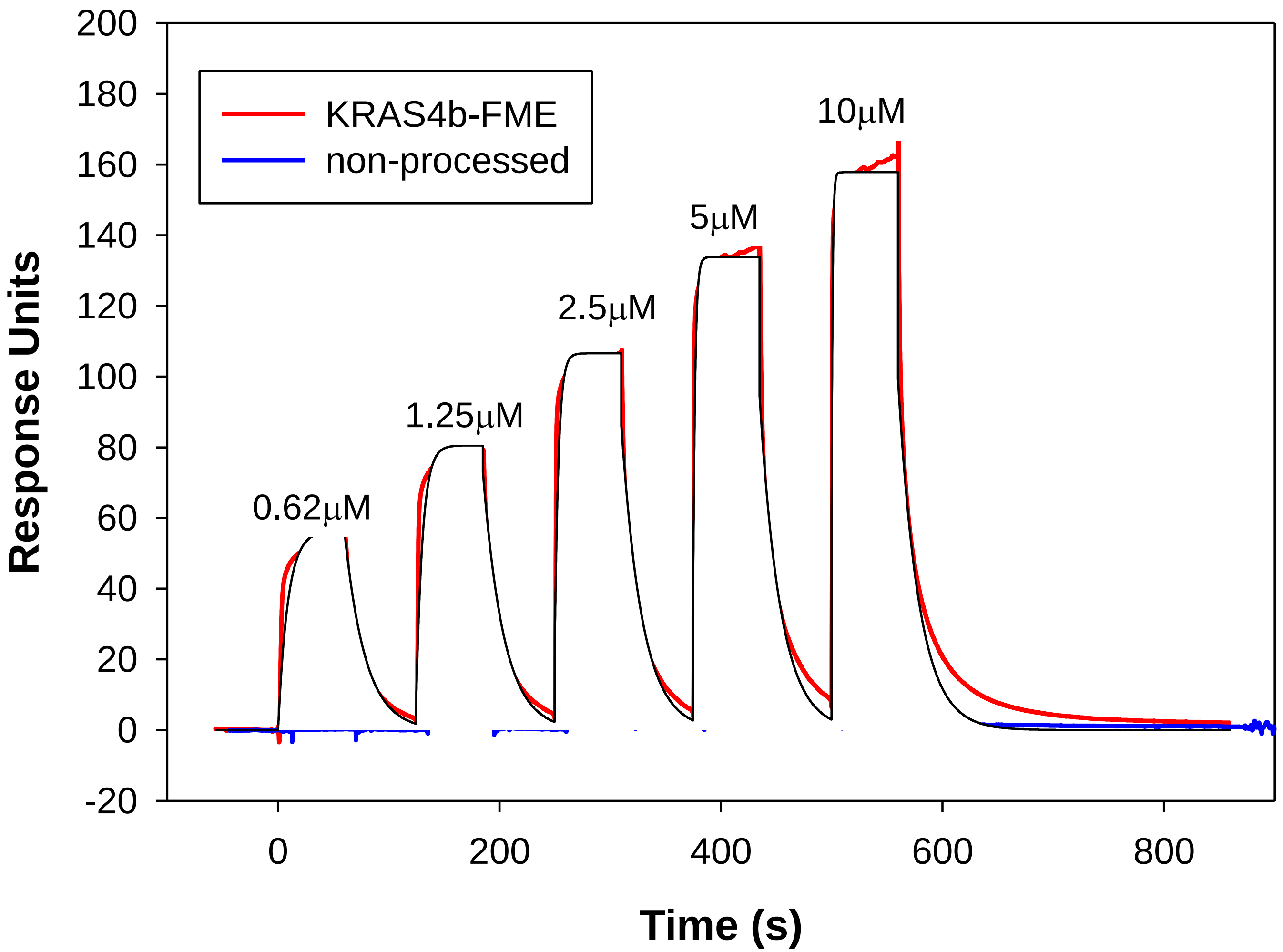

Figure S10: Non-processed KRAS4b does not bind to PDEδ.

Supplementary Figure 1: ESI-MS detection of a mixed population of processed KRAS4b purified from insect cells.
Without separation of the two major forms of processed KRAS4b, a mixed population is obtained (KRAS-FARN, predicted MW of 21412 Da and KRAS4b-FME, predicted MW of 21426 Da).

Supplementary Figure 2: Strategy for engineering the baculovirus genome for prenylated protein production.
Standard AcMNPV bacmid DNA (bMon14272) with a deletion of the chitinase/cathepsin (chi-cath) region was recombineered with a linear substrate (A) containing ≈300 bp homology regions to the chi-cath flanking sequence, a divergent promoter cassette expressing FNTA under the control of the AcMNPV p10 promoter and FNTB under the control of the AcMNPV polyhedrin promoter, and a positive/negative selection marker containing the chloramphenicol acetyltransferase (cat) and sucrose sensitivity (sacB) genes. After recombination and selection, the selection marker can be removed with a 600 bp linear substrate (B) containing flanking homology arms to FNTB and chi-cath as shown. This process creates an intermediate bacmid DNA capable of FNTA/FNTB coexpression. A standard pFastBac style expression clone expressing KRAS can then be transposed into the attTn7 recombination site in the bacmid to create the final bacmid DNA capable of expression of all three proteins.

Supplementary Figure 3: Separation of KRAS-FME from KRAS-FARN using ion exchange chromatography. The mixture of proteins comprising the IMAC pool can be further resolved through cation exchange chromatography. The chromatogram (A280) of the separation reveals the presence of distinct elution peaks (confirmed by the Coomassie-stained SDS-PAGE gel). Isolation of peaks 1-5, followed by further purification (Tev protease digestion, IMAC) and intact mass analysis (Table) enabled identification of the species. Processing of peak 4 resulted in a final sample with a mixture of species. T183* = KRAS4b truncated at T183; FARN = farnesylated KRAS4b; FME = farnesylated and methylated KRAS4b.

Supplementary Figure 4: MALDI-TOF MS/MS analysis of m/z 2199.350. The spectrum information was compared with the expected sequence KMSKDGKKKKKKSKKTKC with farnesyl group attached to terminal Cys (218 mass units). Both N-terminal *b* - and C-terminal *y*–ions were identified.

Supplementary Figure 5: Circular dichroism spectra of non-processed and KRAS4b-FME. CD spectra were collected at 25° C from 250-190 nm with 1mm path length and 0.1mg/ml protein. Thermal denaturation was measured by loss of absorbance at 222 nm with a continuous temperature ramp of 1° C min^{-1}

Supplementary Figure 6: AUC and DLS data indicates KRAS4b-FME is predominantly monomeric. Sedimentation velocity centrifugation of KRAS4b-FME indicates that 86% of the total mass is present with an estimated mass of 22.4 ± 2.4 kDa. (a). Dynamic light scattering analysis of 1 mg/ml KRAS4b-FME indicates 97% is monomeric and 3.3% as larger aggregates.

Supplementary Figure 7: Non-processed KRAS4b does not bind to Nanodiscs. The binding kinetics of non-processed KRAS4b (red) and KRAS4b-FME (black) to 30% DMPS containing Nanodiscs was measured by surface plasmon resonance spectroscopy. Concentrations of non-processed and KRAS4b-FME from 0.93–15 μM were flowed over the nanodisc surface. No binding is observed with non-processed KRAS4b.

Supplementary Figure 8: Farnesylated KRAS4b binding to 30% DMPS Nanodiscs. The binding kinetics of KRAS4b-FARN (non-methylated) to 30% DMPS containing Nanodiscs was measured by surface plasmon resonance spectroscopy (a). Concentrations of KRAS4b-FARN from 1.25 –20 μM in duplicate were flowed over the nanodisc surface. The binding equilibrium were fit assuming a 1:1 binding model (K_D ± SE; 15.0 ± 1.2 μM).

Supplementary Figure 9: KRAS4b binds to CRAF-RBD. Binding of KRAS4b to GST-CRAF-RBD is detected using the alpha assay. The streptavidin conjugated donor bead recognizes the biotinylated Avi-tag on the KRAS4b and transmits a singlet oxygen to the glutathione sepharose acceptor bead that recognizes the GST tag on the CRAF-RBD, resulting in fluorescence at 615 nm. Fluorescence is dependent on concentration of KRAS4b (**a**) and the presence of a GTP analogue (GMP-PNP) bound to KRAS4b (**b**). All data points contain 300 nM GST-CRAF-RBD and 100 nM KRAS4b unless otherwise indicated.

Supplementary Figure 10: Non-processed KRAS4b does not bind to PDEδ.
The binding kinetics of non-processed KRAS4b and KRAS4b-FME to PDE**d** were measured by surface plasmon resonance spectroscopy. PDEδ was amine coupled to a Biacore sensor chip and variable concentrations (0.62–10 mM) of non-processed KRAS4b or KRAS4b-FME were flowed over the surface. The KRAS4b-FME binding curves were fit to a 1:1 binding model shown by the black line.
